# Determinants of Preeclampsia among Women Attending Delivery Services in Public Hospitals of Central Tigray, Northern Ethiopia: A Case-Control Study

**DOI:** 10.1155/2021/4654828

**Published:** 2021-06-01

**Authors:** Teklehaimanot Gereziher Haile, Nega Assefa, Tadesse Alemayehu, Teklewoini Mariye, Gebreamlak Gebremedhn Geberemeskel, Degena Bahrey, Guesh Mebrahtom, Biniyam Demisse, Hailemikael Gebrekidan, Tamirat Getachew

**Affiliations:** ^1^School of Nursing, College of Health Sciences, Axum University, Tigray, Ethiopia; ^2^School of Public Health, College of Health and Medical Sciences, Haramaya University, Harar, Ethiopia; ^3^School of Nursing, Department of Adult Health Nursing, Axum University, Tigray, Ethiopia; ^4^School of Nursing, Department of Pediatrics and Child Health Nursing, Axum University, Tigray, Ethiopia; ^5^School of Nursing and Midwifery, College of Health and Medical Sciences, Haramaya University, Harar, Ethiopia

## Abstract

**Background:**

Preeclampsia occurs in up to 5% of all pregnancies, in 10% of first pregnancies, and 20–25% of women with a history of chronic hypertension.

**Objective:**

This study aims to assess the determinants of preeclampsia among women attending delivery services in public hospitals of central Tigray, Ethiopia.

**Methods:**

Hospital-based unmatched case-control study design was conducted. Women diagnosed with preeclampsia were cases, and women who had no preeclampsia were controls admitted to the same hospitals. A systematic sampling technique was used to select study participants for both cases and controls. The data were entered in EPI data 3.1 statistical software and, then, exported to SPSS Version 22 for cleaning and analysis.

**Results:**

Family history of hypertension (AOR: 2.60; 95% CI: 1.15, 5.92), family history of preeclampsia (AOR: 5.24; 95% CI: 1.85, 14.80), history of diabetes mellitus (AOR: 4.31; 95% CI: 1.66, 11.21), anemia (AOR: 3.23; 95% CI: 1.18, 8.86), history of preeclampsia on prior pregnancy (AOR: 5.55; 95% CI: 1.80, 17.10), primigravida (AOR: 5.41; 95% CI: 2.85, 10.29), drinking alcohol during pregnancy (AOR: 4.06; 95% CI: 2.20, 7.52), and vegetable intake during pregnancy (AOR: 0.39; 95% CI: 0.21, 0.74) were significantly associated with preeclampsia.

**Conclusion:**

This study concludes that a family history of hypertension and preeclampsia; a history of diabetes mellitus and anemia; and a history of preeclampsia on prior pregnancy, primigravida, and drinking alcohol were found to be risk factors for preeclampsia. However, vegetable intake was found to be a protective factor for the development of preeclampsia.

## 1. Introduction

Preeclampsia is a hypertensive condition common to pregnancy that usually occurs after 20 weeks of gestation and affects both the mother and the fetus [1]. It is one of the leading causes for the admission of pregnant women to intensive care units in the world [2]. Annually, it also accounted for about 50,000 maternal mortality worldwide [3].

According to the World Health Organization, the prevalence of preeclampsia is estimated to be seven times higher in developing countries (2.8% of live births) than in developed countries (0.4%) [4], while preeclampsia is the leading cause of high neonatal and maternal mortality in developing countries where maternal resources like prenatal care are scarce, especially in sub-Saharan Africa [5]. Preeclampsia occurs in up to 5% of all pregnancies, in 10% of first pregnancies, and 20–25% of women with a history of chronic hypertension [6].

Preeclampsia leads to adverse health consequences, and it is also costly because of the needed medical services to treat pregnant and postpartum women and their infants, who are often born preterm [7, 8]. In the United States, from the total cost of $2.18 billion found to the health care system, $1.03 billion was in maternal health care and $1.15 billion for infants born from mothers with preeclampsia, and from this, about one-third of the total $6.4 billion short-term estimated health care costs for preeclampsia pregnancies [9].

In Ethiopia, preeclampsia is one of the five major obstetric causes of maternal mortality, and the proportion of maternal mortality from severe preeclampsia or eclampsia shows an increasing trend [10]. Another report from the Felege Hiwot referral hospital indicated that the number of women diagnosed to have preeclampsia was increasing at an alarming rate. For example, from 2012 to 2013, the occurrence of preeclampsia was increased by 83% from 233 to 426 without any change in the diagnosis and reporting system [11].

Identifying the determinants of preeclampsia among women attending delivery services will enable healthcare professionals to successfully tackle its impact on mothers and the fetus. Moreover, it will help health policymakers to design an appropriate strategy to reduce health-associated costs.

So far, there is no established evidence on the determinants of preeclampsia among pregnant women in Tigray, Northern Ethiopia. Hence, this study was aimed to assess the determinants of preeclampsia among women attending delivery services in public hospitals of central Tigray, Northern Ethiopia.

## 2. Methods

An unmatched case-control research design based at the hospital was conducted among women attending delivery service in central Tigray, Ethiopia, public hospitals. The Central Zone is one of seven federal zones in the state of Tigray. There are three general public hospitals in this area (Saint Marry General Hospital, Adwa General Hospital, and Abyi Adi General Hospital) and one full specialized hospital (Axum University comprehensive specialized Hospital).

EPI info software version 7.1.1 was used to calculate the sample size using the double population proportion formula to estimate the sample size required for an unmatched case-control study. The following assumptions were considered to estimate the required sample size for the study: a 95% confidence level, 80% power, primigravida as a risk factor with a lowest odds ratio of 2.16 [12], the proportion of controls with exposure 39%, and the proportion of cases with exposure 58%. Case to control the ratio of 1 : 3 was employed. The final estimated sample size with assuming of 10% nonresponse rate was 344 with 86 cases and 258 controls.

The selection of study participants was made using a systematic sampling technique. *K*, for cases and controls, was calculated by dividing the total number of cases and controls (*N*) to the total samples of (*n*) of cases and controls, respectively. This was computed for each selected hospital. Using the *K* value, the patients were selected in every *K* interval for cases and controls, and the first study subject was selected by the lottery method.

The questionnaire has been adapted from various related literature including preeclampsia-related studies from the WHO and EDHS. Data were collected using interviewer-administered semistructured, pretested questionnaires. Additionally, a record review of participants was conducted to identify cases and controls from the client's registry. After cases and controls were differentiated, data were collected from record review cards and interviews of the study participants. Continuous follow-up and supervision were made by the supervisors and principal investigator throughout the data collection period. All participants were evaluated and privately interviewed. Preeclampsia was the dependent variable, and the independent variables were sociodemographic factors, medical disease factors, obstetric history factors, and maternal behavior factors.

To ensure data quality, the questionnaire was initially prepared in English and then translated into the local language (Tigrigna) by an individual with a good two-language ability and, then, translated back into English by an individual with good translation ability to ensure continuity. The training was given by the principal investigator for data collectors and supervisors in Axum town for two days. The questionnaire was pretested on five percent of the total sample size in Suhul General Hospital one week before the actual data collection period. Data were collected by eight BSc trained midwives and two supervisors having a BSc degree in health. The data collected were reviewed and tested at the spot by supervisors and principal investigators on a daily basis for completeness and accuracy.

Data were entered into the EPI Data version 3.1 statistical software and, then, exported to SPSS version 22 for analysis. Descriptive statistics were used to characterize the sample, and numerical data was presented as frequency, proportion, or percentages. Bivariable was used to examine the statistical association between the dependent variable and independent variables. Variables with a *p* value of ≤0.25 in the bivariable analysis were entered into a multivariable logistic regression to isolate an independent effect of the predictors. The Hosmer-Leme show test was used to check the appropriateness of the model for analysis. Multicollinearity was assessed by a variance inflation factor (VIF). Finally, multivariable logistic regression analysis was carried out to evaluate the combined effect of several factors associated with preeclampsia after adjusting for confounding variables. Adjusted odds ratios (AOR) with 95% CI were used to express the magnitude of the effect of each category on the outcome relative to the reference category. *p* value <0.05 was used to determine the level of statistical significance.

## 3. Results

### 3.1. Sociodemographic Factors of Study Participants

In this research, a total of 86 women who had preeclampsia (cases) and 258 women who had no preeclampsia (controls) were completed the interview with a response rate of 100%. Sixty-four (74.4%) of the cases and 200 (77.5%) of the controls were in the age group of 20 to 34 years.

Eighty-five (98.8%) of the cases and 255 (98.8%) of the controls were Tigrayan by ethnicity. Eighty-three (96.5%) cases and 223 (86.4%) controls were orthodox Christians by religion, and fifty-two (60.5%) cases and 175 (67.8%) controls were living in the urban area. Eighty (93.0%) cases and 246 (95.3%) controls were found married or living together with their partner. Concerning the occupational status of study participants, fifty-four (62.8%) cases and 137 (53.1%) controls were found to be a housewife. Regarding the educational status, 32 (37.2%) of the cases and 95 (36.8%) of the controls completed primary school (Table 1).

### 3.2. Medical Disease History of Study Participants

Of the total participants, 15 (17.4%) cases and 26 (10.1%) controls had a family history of hypertension, and 7 (8.1%) cases and 6 (2.3%) controls had a family history of diabetes mellitus. Thirteen (15.1%) cases and 12 (4.7%) controls had a family history of preeclampsia. Eleven (12.8%) cases and 16 (6.2%) controls were found having a history of diabetes mellitus, while 10 (11.6%) cases and 14 (5.4%) controls were found having anemia. Nine (10.5%) cases and 14 (5.4%) controls had a history of preeclampsia on their prior pregnancy (Table 2).

### 3.3. Obstetric History Factors of Study Participants

Of the total participants, 50 (58.1%) of the cases and 88 (34.1%) of the controls were primigravida, and 30 (83.3%) cases and 141 (82.9%) controls were found with interpregnancy interval <3 years. Seventy-four (86.0%) cases and 235 (91.1%) controls reported that their pregnancy was planned. Eighty-one (94.2%) cases and 251 (97.3%) controls had singleton birth. Of the total participants, 52 (60.5%) of the cases and 137 (53.1%) of the controls had a male baby (Table 3).

### 3.4. Pregnancy Behavior Factors of Study Participants

Of the total participants, 38 (44.2%) cases and 51 (19.8%) controls reported that they drink alcohol during their current pregnancy. Eighty (93.0%) of the cases and 247 (95.7%) of the controls attended ANC at least once for their current pregnancy, while 5 (6.3%) of the cases and 34 (13.8%) of the controls had 4 and more visits. Forty-seven (54.7%) of the cases and 177 (68.6%) of the controls were using modern contraceptives before this pregnancy. Fifty-eight (67.4%) of the cases and 214 (82.9%) of the controls reported to have had fruit intake during this pregnancy, and 56 (65.1%) cases and 212 (82.2%) controls reported they used vegetable (Table 4).

After considering all assumptions of binary logistic regression, those variables which had *p* value ≤ 0.25 at bivariable analysis entered into multivariable logistic regression. In the multivariable logistic regression analysis, eight variables are identified as determinants of preeclampsia among women attending delivery services at a 5% level of significance.

The multivariable analysis revealed that women who had a family history of hypertension had 2.60 times higher risk of preeclampsia compared to women who had no family history of hypertension (AOR: 2.60 at 95% CI: 1.15, 5.92). Those who had a history of DM had 4.31 times higher risk of developing preeclampsia compared to women who had no history of DM (AOR:4.31 at 95% CI: 1.66, 11.21).

The pregnant women who had a family history of preeclampsia were 5.24 times more likely to develop preeclampsia than those who had no family history of preeclampsia (AOR: 5.24 at 95% CI: 1.85, 14.80). Women who had anemia also showed a relationship with preeclampsia among pregnant women. The odd of developing preeclampsia was 3.23 times associated with those who had anemia than those who had no anemia (AOR: 3.23 at 95% CI: 1.18, 8.86).

The participants with a history of preeclampsia on their prior pregnancy were strongly associated with preeclampsia development. The odds of developing preeclampsia were 5.55 times higher for women with a history of preeclampsia comparing to women who had no history of preeclampsia (AOR: 5.55 at 95% CI: 1.80, 17.10).

From factors related to the obstetric history of pregnant women, primigravida was found to be a risk factor for preeclampsia on multivariable analysis. The odds of developing preeclampsia were 5.41 times higher in women with primigravida comparing to the women with multigravida (AOR: 5.41 at 95% CI: 2.85, 10.29).

In the multivariable analysis, women who reported drinking alcohol during their pregnancy period had an increased risk of preeclampsia as compared to those women who did not drink alcohol (AOR: 4.06 at 95%CI = 2.20, 7.52). Women who reported to have taken fruit during pregnancy were found to be protective of preeclampsia in the bivariable analysis. But, the effect did not remain significant after adjusting for potential confounding variables while running the last model. However, women who reported taking vegetables during pregnancy were found to be a protective factor for preeclampsia in the multivariable analysis (Table 5).

## 4. Discussion

The study provides information about the determinants of preeclampsia among pregnant women attending delivery service in public hospitals in Central Tigray, Ethiopia. This study found that a family history of hypertension; a family history of preeclampsia; a history of DM, anemia, and primigravida; a history of preeclampsia on prior pregnancy; and alcohol drinking during pregnancy are risk factors, whereas taking vegetable during pregnancy was a protective factor for developing preeclampsia.

Those who had a family history of hypertension were significantly associated with preeclampsia among pregnant women. The odds of developing preeclampsia were 2.60 times more than those who had no family history of hypertension. This finding is also consistent with other studies conducted in Ethiopia, Uganda, and South India [13–15].

History of DM showed a significant association with preeclampsia among pregnant women than those who had no history of DM. Those who had a history of DM were 4.31 times more related to developing preeclampsia compared to their counterparts. This finding is supported by different studies conducted in different countries; such as South India, Gaza Strip, and Ethiopia [13, 15, 16]. This is explained by epidemiological and clinical data document has shown that DM is closely associated with insulin resistance [17]. Besides, hyperinsulinemia is stimulating the proliferation of vascular smooth muscle cells, enhances acute sympathetic nervous system activity, and modifies transmembrane cation transport, as well as renal sodium retention, the release of the potent vasoconstrictor angiotensin II, and associated endothelial dysfunction. All of these alterations may contribute to blood pressure elevation and thus preeclampsia [18].

In this study, the multivariable analysis revealed that a family history of preeclampsia was significantly associated with preeclampsia development. Women who had a family history of preeclampsia were 5.24 times higher odds of developing preeclampsia than their counterparts. This finding is in agreement with the study conducted in India and Yemen [19, 20]. This might have occurred because of genetic and/or behavioral factors that contribute to the pathophysiologic susceptibility of preeclampsia that cluster in families [21].

The participants diagnosed with anemia were significantly associated with preeclampsia development. The odds of developing preeclampsia among women diagnosed with anemia is 3.23 times more than women who had no anemia. Similarly, other studies conducted in India and Ethiopia reported that anemia was significantly associated with preeclampsia [13, 19]. Contrary to this, the study conducted in Iran showed that anemia was protective for preeclampsia [22]. This difference could be due to variation in sample size and study participants. The participants in the Iranian study were excluded those who are over 35 years of age or below 18 years of age, history of any maternal disease including diabetes unlike this study.

Those participants who had a history of preeclampsia on prior pregnancy had 5.55 times higher risk of developing preeclampsia than their counterparts. This finding is supported by studies conducted in the Gaza Strip, Yemen, and Ethiopia [16, 20, 23]. This showed that women with a history of preeclampsia on their previous pregnancy need to have a focus and could be an acceptable means of screening for preeclampsia, especially in limited resourced locations.

This study showed that primigravida was found independently associated with preeclampsia development. The odds of developing preeclampsia in primigravida women were 5.41 times higher than multigravida women. This finding is consistent with studies conducted in the Gaza Strip, Egypt, and Ethiopia [16, 23, 24], as they declared that primigravida was a risk factor for preeclampsia. Preeclampsia generally considered a disease of the first pregnancy [25] which is due to the immunological incompetence seen in the first pregnancy between fetoplacental and maternal tissues [26].

Alcohol consumption also showed an association with preeclampsia development among pregnant women. The odds of developing preeclampsia were 4.06 times more common among women who had drink alcohol than those who did not drink alcohol. This finding is consistent with a study conducted in other areas of Ethiopia [23, 27]. In contrast to this finding, a study conducted in India [28] was found that no significant association between alcohol drinking during pregnancy and preeclampsia. Unlike the current study, a cross-sectional study conducted in India was based on the women's report of clinical manifestation for preeclampsia and was not diagnosed by physicians. The discrepancy could be due to the difference in study design and study subjects.

Those participants who reported consumption of vegetables had a 61% lower risk of developing preeclampsia than their counterparts. This finding is supported by other studies conducted in Ethiopia [23, 29]. This is because vegetables are rich in micronutrients such as antioxidants, vitamins, minerals, and dietary fiber. A diet rich in vegetables decreased the risk of hyperhomocysteinemia, which is one of the risk factors for the occurrence of preeclampsia [30].

## 5. Conclusion

The results of this study suggest that there are protective and risk factors for preeclampsia. Factors such as a family history of hypertension, family history of preeclampsia, history of DM, diagnosed with anemia, and history of preeclampsia on prior pregnancy, primigravida, and drinking alcohol during pregnancy were found to be risk factors for preeclampsia. However, vegetable intake during pregnancy was found to be a protective factor for the development of preeclampsia.

## Figures and Tables

**Table 1:** Sociodemographic factors of women attending delivery service in public hospitals in the central zone, Tigray, Northern Ethiopia, 2019.

Variables	Category	Case	Control
		Number (%)	Number (%)
Age	<20	9 (10.5%)	26 (10.1%)
	20-34	64 (74.4%)	200 (77.5%)
	≥35	13 (15.1%)	32 (12.4%)

Ethnicity	Tigray	85 (98.8%)	255 (98.8%)
	Amhara	1 (1.2%)	3 (1.2%)

Religion	Orthodox	83 (96.5%)	223 (86.4%)
	Muslim	3 (3.5%)	35 (13.6%)

Residence	Urban	52 (60.5%)	175 (67.8%)
	Rural	34 (39.5%)	83 (32.2%)

Occupation	House wife	54 (62.8%)	137 (53.1%)
	Merchant	16 (18.6%)	63 (24.4%)
	Government employee	11 (12.8%)	45 (17.4%)
	Others	5 (5.8%)	13 (5.0%)

Marital status	Never married	6 (7.0%)	6 (2.3%)
	Married/living together	80 (93.0%)	246 (95.3%)
	Separated	0 (0.0%)	6 (2.3%)

Educational status	No formal education	10 (11.6%)	31 (12.0%)
	Primary secondary	32 (37.2%)	95 (36.8%)
	Technical/vocational	24 (27.9%)	79 (30.6%)
	Higher education	15 (17.4%)	25 (9.7%)
		5 (5.8%)	28 (10.9%)

**Table 2:** Disease status of women attending delivery service in public hospitals in the central zone, Tigray, Northern Ethiopia, 2019.

Variables	Category	Cases	Controls
		Number (%)	Number (%)
Family history of hypertension	Yes	15 (17.4%)	26 (10.1%)
	No		

Who had a history of hypertension	Father	71 (82.6%)	232 (89.9%)
	Mother	2 (13.3%)	9 (34.6%)
	Brother	11 (73.3%)	7 (26.9%)
	Sister	2 (13.3%)	6 (23.1%)
		0 (0.0%)	4 (15.4%)

Family history of DM	Yes	7 (8.1%)	6 (2.3%)
	No	79 (91.9%)	252 (97.7%)

Who had a history of DM	Father	3 (42.9%)	2 (33.3%)
	Mother	2 (28.5%)	2 (33.3%))
	Brother	1 (14.3%)	0 (0.0%)
	Sister	1 (14.3%)	2 (33.3%))

Family history of preeclampsia	Yes	13 (15.1%)	12 (4.7%)
	No		
Who had a history of preeclampsia	Mother	73 (84.9%)	246 (95.3%)
	Sister	12 (92.3%)	6 (50.0%)
		1 (7.7%)	6 (50.0%)

History of DM	Yes	11 (12.8%)	16 (6.2%)
	No	75 (87.2%)	242 (93.8%)

Diagnosed with anemia	Yes	10 (11.6%)	14 (5.4%)
	No	76 (88.4%)	244 (94.6%)

History of preeclampsia on prior pregnancy	Yes	9 (10.5%)	14 (5.4%)
	No	77 (89.5%)	244 (94.6%)

**Table 3:** Obstetric history of women attending delivery service in public hospitals in the central zone, Tigray, Northern Ethiopia, 2019.

Variables	Category	Cases	Controls
		Number (%)	Number (%)
Gravidity	Primigravida	50 (58.1%)	88 (34.1%)
	Multigravida	36 (41.9%)	170 (65.9%)

History of abortion	Yes	7 (19.4%)	22 (12.9%)
	No	29 (80.6%)	148 (87.1%)

Number of abortions	1	5 (71.4%)	21 (95.5%)
	≥2	2 (28.6%)	1 (4.5%)

Parity	Nullipara	50 (58.1%)	88 (34.1%)
	One delivery	11 (12.8%)	54 (20.9%)
	Multipara	25 (29.1%)	116 (45.0%)

Planned pregnancy	Yes	74 (86.0%)	235 (91.1%)
	No	12 (14.0%)	23 (8.9%)

Multiplicity of pregnancy	Singleton	81 (94.2%)	251 (97.3%)
	Twin	5 (5.8%)	7 (2.7%)

Sex of newborn	Male	52 (60.5%)	137 (53.1%)
	Female	34 (39.5%)	121 (46.9%)

Pregnancy interval	<3Year	30 (83.3%)	141 (82.9%)
	≥3Year	6 (16.7%)	29 (17.1%)

**Table 4:** Behavior factors of pregnant women attending delivery service in public hospitals in the central zone, Tigray, Northern Ethiopia, 2019.

Variables	Category	Cases	Controls
		Number (%)	Number (%)
Alcohol intake	Yes	38 (44.2%)	51 (19.8%)
	No	48 (55.8%)	207 (80.2%)

Attending ANC	Yes	80 (93.0%)	247 (95.7%)
	No	6 (7.0%)	11 (4.3%)

Frequency of attending ANC	<4	75 (93.8%)	213 (86.2%)
	≥4	5 (6.3%)	34 (13.8%)

Use of modern contraceptive	Yes	47 (54.7%)	177 (68.6%)
	No	39 (45.3%)	81 (31.4%)

Fruit intake	Yes	58 (67.4%)	214 (82.9%)
	No	28 (32.6%)	44 (17.1%)

Frequency of fruit intake per week	Daily	6 (10.3%)	8 (3.7%)
	1-6 days/week	52 (89.7%)	206 (96.3%)

Vegetable intake	Yes	56 (65.1%)	212 (82.2%)
	No	30 (34.9%)	46 (17.8%)

Frequency of vegetable intake per week	Daily	3 (5.4%)	3 (1.4%)
	1-6 days/week	53 (94.6%)	209 (98.6%)

**Table 5:** The bivariable and multivariable analysis of determinants of preeclampsia for women attending delivery service in public hospitals in the central zone, Tigray, Ethiopia, 2019.

Variables	Category	Cases Number (%)	Controls Number (%)	COR (95% CI)	AOR (95%: CI)
Family history of DM	Yes	7 (8.1%)	6 (2.3%)	3.72 (1.21, 11.40)	3.45 (0.92,12.90)
	No	79 (91.9%)	252 (97.7%)	1	1

Use of modern contraceptive	Yes	47 (54.7%)	177 (68.6%)	0.55 (0.33,0.91)	0.65 (0.36,1.17)
	No	39 (45.3%)	81 (31.4%)	1	1

Family history of hypertension	Yes	15 (17.4%)	26 (10.1%)	1.88 (0.95,3.75)	2.60 (1.15,5.92)∗
	No	71 (82.6%)	232 (89.9%)	1	1

Family history of preeclampsia	Yes	13 (15.1%)	12 (4.7%)	3.65 (1.60, 8.35)	5.24 (1.85,14.80)∗
	No	73 (84.9%)	246 (95.3%)	1	1

History of DM	Yes	11 (12.8%)	16 (6.2%)	2.22 (0.99,4.99)	4.31 (1.66,11.21)∗
	No	75 (87.2%)	242 (93.8%)	1	1

Diagnosed with anemia	Yes	10 (11.6%)	14 (5.4%)	2.29 (0.98, 5.37)	3.23 (1.18,8.86)∗
	No	76 (88.4%)	244 (94.6%)	1	1

History of preeclampsia on prior pregnancy	Yes	9 (10.5%)	14 (5.4%)	2.04 (0.85, 4.89)	5.55 (1.80,17.10)∗
	No	77 (89.5%)	244 (94.6%)	1	1

Gravidity	Primigravida	50 (58.1%)	88 (34.1%)	2.68 (1.63, 4.42)	5.41 (2.85,10.29)∗∗
	Multigravida	36 (41.9%)	170 (65.9%)	1	1

Alcohol drinking	Yes	38 (44.2%)	51 (19.8%)	3.21 (1.90, 5.43)	4.06 (2.20,7.52)∗∗
	No	48 (55.8%)	207 (80.2%)	1	1

Fruit intake	Yes	58 (67.4%)	214 (82.9%)	0.43 (0.24, 0.74)	0.89 (0.37,2.10)
	No	28 (32.6%)	44 (17.1%)	1	1

Vegetable intake	Yes	56 (65.1%)	212 (82.2%)	0.40 (0.23,0.70)	0.39 (0.21,0.74)∗
	No	30 (34.9%)	46 (17.8%)	1	1

NB: ^∗^*p* value < 0.05; ^∗∗^*p*-value < 0.001.

## Data Availability

This is our original work and has not been submitted and considered for publish in any journal and all sources of materials and data used for this research have been secured and acknowledged. All raw data generated or analyzed during the current study are available from the corresponding author on request.

## Abbreviations

ANC: Antenatal care
AOR: Adjusted odds ratio
COR: Crude odds ratio
DM: Diabetes mellitus.
